# Disparities in Utilization of Uterine Fibroid Embolization

**DOI:** 10.1001/jamanetworkopen.2025.32100

**Published:** 2025-09-16

**Authors:** Tarig S. Elhakim, Sara Smolinski-Zhao, Dominie Miyasato, Vladlena Lee, Arian Mansur, Maria Puello, Nathaniel Mercaldo, Anna Sophia McKenney, Sanjeeva P. Kalva, Michael Dezube, Dania Daye

**Affiliations:** 1 Perelman School of Medicine at the University of Pennsylvania, Philadelphia; 2 Department of Radiology, Massachusetts General Hospital, Boston; 3 Department of Radiology, Harvard Medical School, Boston, Massachusetts; 4 School of Medicine, Universidad Del Norte, Atlantico, Colombia; 5 Department of Radiology, New York–Presbyterian Hospital/Weill Cornell Medical Center, New York, New York

## Abstract

**Importance:**

Uterine fibroid embolization (UFE) is a minimally invasive alternative to surgery. Understanding utilization patterns and disparities in access is important to ensure that patients can explore all treatment options.

**Objective:**

To examine trends in the use of UFE vs hysterectomy and myomectomy for uterine fibroid management, with an emphasis on sociodemographic and institutional disparities.

**Design, Setting, and Participants:**

This cross-sectional analysis used data from the 2016 to 2022 National Inpatient Sample obtained from the Healthcare Cost and Utilization Project, a population-based, multicenter inpatient dataset representing hospitals across the US. Adult patients with a diagnosis of uterine fibroids who underwent hysterectomy, myomectomy, or UFE were identified using *International Statistical Classification of Diseases, Tenth Revision, Clinical Modification* codes. Analysis was conducted in April 2025.

**Exposure:**

Patient age, race, ethnicity, insurance, income quartile, rurality, year of procedure, and hospital characteristics.

**Main Outcomes and Measures:**

The primary outcome was undergoing UFE, modeled using multivariable logistic regression, with hysterectomy, myomectomy, or surgery overall as reference groups. The covariate reference categories were age younger than 30 years, White race, private insurance, 76th to 100th income percentile, central metropolitan residence, the year 2016, small hospitals, rural hospitals, and hospitals in the Pacific division. Results were reported as adjusted odds ratios (aORs) with 95% CIs.

**Results:**

The sample encompassed 271 885 encounters, including 199 625 hysterectomies (73.4%), 62 675 myomectomies (23.1%), and 9585 UFEs (3.5%). The median (IQR) patient age was 47 (43-52) years for those undergoing hysterectomy, 45 (40-49) years for those undergoing UFE, and 37 (33-41) years for those undergoing myomectomy. With regard to race and ethnicity, 105 780 patients (38.9%) were African American, 16 175 (5.9%) were Asian or Pacific Islander, 48 810 (18.0%) were Hispanic, 1050 (0.4%) were Native American, 86 425 were White (31.8%), and 13 645 (5.0%) were other races. Increasing age was associated with lower odds of undergoing UFE vs hysterectomy, and higher odds of undergoing UFE vs myomectomy. African American patients were more likely to undergo UFE than hysterectomy (aOR, 1.64; 95% CI, 1.44-1.87), but less likely to undergo UFE than myomectomy (aOR, 0.84; 95% CI, 0.73-0.97). Hispanic patients were less likely to undergo UFE than both surgical procedures (aOR, 0.83; 95% CI, 0.71-0.97). Patients with Medicaid (aOR, 1.58; 95% CI, 1.41-1.77), self-pay (aOR, 1.97; 95% CI, 1.60-2.42), and no-charge (aOR, 1.97; 95% CI, 1.24-3.12) coverage had higher odds of undergoing UFE vs both surgical procedures. Among Medicare patients, UFE was more likely than myomectomy among those aged 30 to 49 years, but less likely among those aged 50 years and older. Those in the lowest income quartile (0-25th percentile) had greater odds of undergoing UFE vs myomectomy (aOR, 1.22; 95% CI, 1.04-1.43). Rural patients were less likely to undergo UFE than hysterectomy (aOR, 0.53; 95% CI, 0.34-0.83), whereas urban hospitals were more likely to perform UFE than both surgical procedures (aOR, 7.13; 95% CI, 3.43-14.80).

**Conclusions and Relevance:**

In this cross-sectional study, UFE was underutilized with significant disparities across socioeconomic factors. Further efforts are needed to equitably expand access to UFE across the country.

## Key Points

**Question:**

What are the national utilization patterns and disparities in access to uterine fibroid embolization (UFE) compared with hysterectomy and myomectomy?

**Findings:**

In this cross-sectional study of 271 885 uterine fibroid encounters, UFE was underutilized (3.5%) compared with hysterectomy (73.4%) and myomectomy (23.1%). Hysterectomy was more common among White, Hispanic, and privately insured patients and in rural areas, whereas UFE was more commonly performed among African American patients, those without private insurance, and those in lower-income communities.

**Meaning:**

These finding suggest that there are substantial disparities in UFE utilization and highlight the need for targeted policies and referral strategies to promote equitable access to minimally invasive fibroid treatments.

## Teaser

This cross-sectional study examines national utilization patterns and disparities in access to uterine fibroid embolization compared with hysterectomy and myomectomy in the US.

## Introduction

Uterine fibroids are the most prevalent benign tumors in women.^1^ They can cause menorrhagia, pelvic pain, urinary symptoms, infertility, spontaneous abortion, and preterm delivery.^2^ Management options include pharmacologic treatments, minimally invasive procedures, and surgery with myomectomy or hysterectomy. Although a stepwise approach beginning with noninvasive treatment is advised, hysterectomy continues to be predominantly offered.^1,3^ Although it is a definitive treatment, it is also the most invasive, resulting in infertility.^4^ Myomectomy is a uterine-sparing procedure that surgically removes fibroids. Although it reduces heavy bleeding and may conserve fertility, adverse events include myometrial trauma and adhesions.^5^ Both myomectomy and hysterectomy are surgical treatments for uterine conditions performed by gynecologic surgeons. As an alternative to surgery, uterine fibroid embolization (UFE) is a minimally invasive procedure that emerged in the 1990s and has gained popularity because of its safety and efficacy. It is performed by interventional radiologists (IRs), who are physicians trained in image-guided, minimally invasive procedures. Compared with surgery, UFE is a shorter procedure, does not require general anesthesia, has faster recovery, has fewer complications, and costs less.^6^ It can achieve a 42% reduction in fibroid size in 3 months with improvement of symptoms while potentially preserving fertility.^1,3,6,7^ Despite these advantages, UFE remains significantly less utilized than surgery.^7,8^

There is limited evidence about the utilization patterns of fibroid interventions. This gap was outlined by the Agency for Healthcare Research and Quality, prompting the need for investigation to address any disparities.^9^ One study examining outpatient trends found that hysterectomy utilization for fibroids has increased since 2005, while UFE is underutilized.^7^ Another study found that younger patients and African American patients were more likely to undergo UFE.^10^ The purpose of this study is to perform a 7-year longitudinal nationwide analysis of UFE compared with hysterectomy and myomectomy for uterine fibroid management using the largest US database to assess disparities in access across the country, guide policy interventions, and promote equity.

## Methods

### Database Source

This study was exempt from institutional review board approval and the need for informed consent as it involved deidentified, public data, in accordance with 45 CFR §46. The Strengthening the Reporting of Observational Studies in Epidemiology (STROBE) reporting guidelines for cross-sectional studies were used. This study utilized the 2016 to 2022 National Inpatient Sample obtained from the Healthcare Cost and Utilization Project (HCUP), a family of health care databases developed by federal, state, and industry organizations and sponsored by Agency for Healthcare Research and Quality.^11,12^ The *International Statistical Classification of Diseases, Tenth Revision, Clinical Modification* diagnostic codes were used to define the uterine fibroid population (eTable 1 in Supplement 1). This was filtered to those treated with UFE, myomectomy, or hysterectomy using the *International Statistical Classification of Diseases and Related Health Problems, Tenth Revision* Procedure Coding System. The primary and secondary codes were searched anywhere in the database. Myomectomy and hysterectomy interventions were analyzed separately and then combined into a surgical intervention category for comparison with UFE.

### Patient Sample

Sample weighting was performed following National Inpatient Sample guidelines.^13^ The study population began with 645 530 encounters and was refined by excluding records with missing demographic or hospital-level variables (4%) and restricting to hospitals offering all 3 procedures. The analysis was restricted to hospitals with at least 5 admissions for each procedure. Importantly, this removed hospitals that did not offer UFE but offered myomectomy and/or hysterectomy. This helped reduce bias that might otherwise exist by virtue of population settlement in rural areas.

### Study Outcomes and Exposures

The primary outcome was UFE utilization compared with hysterectomy, myomectomy, or surgical intervention. Patient characteristics, including age, race, ethnicity, insurance, and median household income, were assessed. Race and ethnicity data were classified by hospitals using self-report or administrative records. Categories were defined by HCUP (African American, Asian or Pacific Islander, Hispanic, Native American, White, and other, which includes multiracial or unspecified groups) and are included here to assess disparities. The study included the year of procedural intervention and patient’s location using the urban-rural code from the National Center for Health Statistics.^14^ This explores the association between health outcomes and urbanization level, allowing evaluation of health disparities between urban-rural populations. In addition, hospital division, bed size, and teaching status were analyzed.

### Statistical Analysis

Patients’ demographics and hospital characteristics were summarized using descriptive measures. The χ^2^ test with Rao and Scott second-order correction was used for categorical variables to account for the complex survey design,^15^ whereas the Wilcoxon rank-sum test was used for continuous variables. A survey-weighted multivariable logistic regression model was constructed to evaluate the association of different factors with the likelihood of UFE compared with hysterectomy or myomectomy. Separate models were constructed to compare UFE vs hysterectomy, UFE vs myomectomy, and UFE vs hysterectomy or myomectomy. In each, UFE was the outcome of interest to assess factors associated with undergoing UFE vs surgery. The exposures included patient age group (categorized as <30, 30-39, 40-49, and ≥50 years), race, ethnicity, insurance (Medicaid, Medicare, private, self-pay, no charge, and other), zip code–based median household income quartile, urban-rural classification, year of procedure, and hospital characteristics. The reference categories were age younger than 30 years, White race, private insurance, 76th to 100th income percentile, central metropolitan area, year 2016, small or rural hospitals, and hospitals in the Pacific division. The analysis used mutually adjusted logistic regression models, where all exposures were included simultaneously to ensure that the association between any exposure and the outcome accounts for the influence of other covariates. Results were reported as adjusted odds ratios (aORs) with 95% CIs, and 2-sided *P* < .05 was considered statistically significant. Analysis was done using R statistical software version 4.3.1 (R Project for Statistical Computing) and Python software version 3.12.1 (Python Software Foundation).

In age-stratified sensitivity analysis, to evaluate whether differences in UFE utilization by race and insurance varied across reproductive years, a stratified multivariable logistic regression analysis by age group was conducted. Patients were grouped into 4 age categories, and the odds of UFE vs surgical interventions were assessed.

## Results

### Patient Demographics

**Figure 1.**
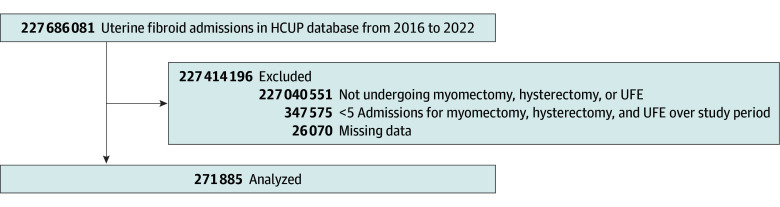
Patient Enrollment Flowchart Patients undergoing treatment for uterine fibroids were initially identified from the Healthcare Cost and Utilization Project (HCUP) national dataset. Exclusion criteria included individuals with missing demographic or hospital data and fewer than 5 admissions for each procedure. The final sample included patients who underwent hysterectomy, myomectomy, or uterine fibroid embolization (UFE) for uterine fibroid management.

A total of 271 885 encounters were included (Figure 1). Of the total cohort, 262 300 patients (96.5%) underwent surgical management, including hysterectomy (199 625 patients [73.4%]) and myomectomy (62 675 patients [23.1%]), whereas 9585 patients (3.5%) underwent UFE (eFigure 1 in Supplement 1). Patients’ ages spanned from 18 to 90 years, with the majority aged 30 to 60 years. The median (IQR) patient age was 47 (43-52) years for those undergoing hysterectomy, 45 (40-49) years for those undergoing UFE, and 37 (33-41) years for those undergoing myomectomy (Table 1). The study had a wide diversity of racial and ethnic backgrounds, with similar proportions of African American (105 780 patients [38.9%]) and White (86 425 patients [31.8%]) patients. In addition, 16 175 patients (5.9%) were Asian or Pacific Islander, 48 810 (18.0%) were Hispanic, 1050 (0.4%) were Native American, and 13 645 (5.0%) were other races. Most were in central areas or outskirts of metropolitan regions (197 160 patients [72.5%]), with a few from micropolitan (10 540 patients [3.9%]) or rural (6600 patients [2.4%]) areas. Most had private insurance (177 725 patients [65.4%]), followed by Medicaid (54 275 patients [20.0%), Medicare (20 415 patients [7.5%]), self-pay (9875 patients [3.6%]), and no-charge (1850 patients [0.7%]). Private insurance was more utilized by White patients (61 880 patients [71.6%]) and Asian or Pacific Islander patients (11 623 patients [71.9%]) (eFigure 2 in Supplement 1). Hispanic patients had the highest coverage by Medicaid (16 498 patients [33.8%]), self-pay (3173 patients [6.5%]), and no-charge (781 patients [1.6%]), while Medicare was highest among Native American (80 patients [7.6%]) and White (9680 patients [11.2%]) patients.

**Table 1.** Patient Characteristics

Characteristic	Patients, No. (%)				*P* value
	Overall (N = 271 885)	Hysterectomy (n = 199 625)	Myomectomy (n = 62 675)	UFE (n = 9585)	
Patient age, y					
Median (IQR)	45 (39-50)	47 (43-52)	37 (33-41)	45 (40-49)	<.001
Age range					
<30	7445 (2.7)	865 (0.4)	6350 (10.1)	230 (2.4)	
30-39	62 090 (22.8)	23 805 (11.9)	36 140 (57.7)	2145 (22.4)	
40-49	128 690 (47.3)	105 865 (53.0)	17 695 (28.2)	5130 (53.5)	
≥50	73 660 (27.1)	69 090 (34.6)	2490 (4.0)	2080 (21.7)	
Race and ethnicity					
African American	105 780 (38.9)	69 430 (34.8)	31 875 (50.9)	4475 (46.7)	<.001
Asian or Pacific Islander	16 175 (5.9)	11 365 (5.7)	4245 (6.8)	565 (5.9)	
Hispanic	48 810 (18.0)	37 930 (119.0)	9365 (14.9)	1515 (15.8)	
Native American	1050 (0.4)	835 (0.4)	175 (0.3)	40 (0.4)	
White	86 425 (31.8)	70 900 (35.5)	13 015 (20.8)	2510 (26.2)	
Other^a^	13 645 (5.0)	9165 (4.6)	4000 (6.4)	480 (5.0)	
Median household income					
76-100th Percentile	67 985 (25.0)	48 205 (224.1)	17 435 (227.8)	2345 (24.5)	<.001
51-75th Percentile	68 180 (25.1)	49 550 (24.8)	16 340 (26.1)	2290 (23.9).	
26-50th Percentile	61 365 (22.6)	45 675 (22.9)	13 555 (21.6)	2135 (22.3)	
0-25th Percentile	74 355 (27.3)	56 195 (28.2)	15 345 (24.5)	2815 (29.4)	
Patient location					
Central counties of metropolitan areas of ≥1 million population	117 390 (43.2)	80 975 (40.6)	31 690 (50.6)	4725 (49.3)	<.001
Fringe counties of metropolitan areas of ≥1 million population	79 770 (29.3)	57 560 (28.8)	19 640 (31.3)	2570 (26.)	
Counties in metropolitan areas of 250 000-999 999 population	45 060 (16.6)	35 815 (17.9)	7650 (12.2)	1595 (16.6)	
Counties in metropolitan areas of 50 000-249 999 populations	12 525 (4.6)	10 325 (5.2)	1875 (3.0)	325 (3.4)	
Micropolitan counties	10 540 (3.9)	9140 (4.6)	1145 (1.8)	255 (2.7)	
Not metropolitan or micropolitan counties	6600 (2.4)	5810 (2.9)	675 (1.1)	115 (1.2)	
Year					
2016	45 985 (16.9)	35 505 (17.8)	9170 (14.6)	1310 (13.7)	<.001
2017	45 015 (16.6)	34 205 (17.1)	9460 (15.1)	1350 (14.1)	
2018	41 320 (15.2)	30 240 (15.1)	9815 (15.7)	1265 (13.2)	
2019	40 495 (14.9)	29 305 (14.7)	9740 (15.5)	1460 (15.1)	
2020	31 445 (11.6)	22 695 105 (11.4)	7515 (12.0)	1235 (12.0)	
2021	35 275 (13.0)	25 160 (12.6)	8705 (13.9)	1410 (14.7)	
2022	32 350 (11.9)	22 515 (11.3)	8270 (13.2)	1565 (16.5)	
Insurance					
Private	177 725 (65.4)	127 025 (63.6)	45 320 (72.3)	5380 (56.1)	<.001
Medicaid	54 275 (20.0)	39 665 (19.9)	12 025 (19.2)	2585 (27.0)	
Medicare	20 415 (7.5)	18 360 (9.2)	1420 (2.3)	625 (6.6)	
No charge	1850 (0.7)	1145 (0.7)	305 (0.5)	100 (1.0)	
Self-pay	9875 (3.6)	7360 (3.7)	1950 (3.1)	565 (5.9)	
Other	7745 (2.8)	5770 (2.9)	1655 (2.6)	320 (3.3)	
Hospital division					
East South Central	9505 (3.5)	7745 (3.9)	1365 (2.2)	395 (4.1)	<.001
East North Central	26 420 (9.7)	20 645 (10.3)	4730 (7.5)	1045 (10.9)	
Middle Atlantic	56 990 (21.0)	39 330 (19.7)	15 765 (25.2	1895 (19.8)	
Mountain	8685 (3.2)	6715 (3.4)	1475 (2.4)	495 (5.2)	
New England	12 030 (4.4)	8340 (4.2)	3140 (5.0)	550 (5.7)	
Pacific	56 660 (20.8)	42 800121.4)	11 870 (18.9)	1990 (20.8)	
South Atlantic	81 300 (29.9)	58 920 (29.5)	19 940 (31.8)	2440 (25.5)	
West North Central	3900 (1.4)	2775 (1.4)	855 (1.4)	270 (2.8)	
West South Central	16 395 (6.0)	12 355 (6.2)	3535 (5.6)	505 (5.3)	
Teaching status					
Rural	7715 (2.8)	7105 (3.6)	570 (0.9)	40 (0.4)	<.001
Urban nonteaching	42 500 (15.6)	33 500 (16.8)	7915 (12.6)	1085 (11.3)	
Urban teaching	221 670 (81.5)	159 020 (79.7)	54 190 (86.5)	8460 (88.3	
Hospital bed size					
Small	37 640 (13.8)	28 190 (14.1)	8205 (13,1)	1245 (13.0)	<.001
Medium	73 750 (27.1)	54 475 (27.3)	16 920 (27.0)	2355 (24.6)	
Large	160 495 (59.0)	116 960 (58.6)	37 550 (59.9)	5985 (62.4)	

Abbreviation: UFE, uterine fibroid embolization.

^a^ Other includes multiracial, unspecified, and groups not categorized under the predefined classification.

### Multivariable Analysis of Patient-Specific Factors

Age groups showed divergent trends in UFE utilization, with increasing age associated with lower odds of UFE over hysterectomy, and higher odds of UFE over myomectomy (Table 2). Compared with White patients, African American patients were more likely to undergo UFE than hysterectomy (aOR, 1.64; 95% CI, 1.44-1.87), but less likely to undergo UFE than myomectomy (aOR, 0.84; 95% CI, 0.73-0.97). Among Hispanic patients, UFE utilization was lower than that for surgical interventions (aOR, 0.83; 95% CI, 0.71-0.97).

**Table 2.** Multivariable Logistic Regression, Stratified by UFE vs Surgical Intervention

Characteristic	UFE vs combined surgical interventions		UFE vs hysterectomy		UFE vs myomectomy	
	aOR (95% CI)	*P* value	aOR (95% CI)	*P* value	aOR (95% CI)	*P* value
Age range, y (reference, <30 y)						
30-39	1.18 (0.86-1.60)	.31	0.35 (0.24-0.49)	<.001	1.77 (1.29-2.42)	<.001
40-49	1.46 (1.08-1.98)	.01	0.19 (0.13-0.27)	<.001	8.89 (6.54-12.10)	<.001
≥50	1.01 (0.74-1.39)	.94	0.12 (0.08-0.17)	<.001	22.50 (16.10-31.40)	<.001
Race and ethnicity (reference, White)						
Asian or Pacific Islander	1.06 (0.86-1.32)	.58	1.17 (0.94-1.46)	.16	0.83 (0.65-1.07)	.15
African American	1.37 (1.21-1.56)	<.001	1.64 (1.44-1.87)	<.001	0.84 (0.73-0.97)	.02
Hispanic	0.83 (0.71-0.97)	.02	0.83 (0.71-0.98)	.02	0.79 (0.66-0.95)	.01
Native American	1.13 (0.56-2.30)	.73	1.14 (0.55-2.36)	.72	0.90 (0.37-2.15)	.81
Other^a^	1.05 (0.83-1.32)	.70	1.16 (0.92-1.47)	.20	0.71 (0.55-0.92)	.009
Median annual household income (reference, 76th-100th percentile)						
51st-75th Percentile	0.92 (0.81-1.06)	.25	0.90 (0.78-1.03)	.12	1.03 (0.88-1.19)	.73
26th-50th Percentile	0.96 (0.83-1.11)	.59	0.93 (0.80-1.07)	.30	1.13 (0.97-1.33)	.13
0-25th Percentile	0.98 (0.85-1.13)	.61	0.88(0.76-1.02)	.09	1.22 (1.04-1.43)	.02
Patient location (reference, central counties of metropolitan areas of ≥1 million population)						
Fringe counties of metropolitan areas of ≥1 million population	0.86 (0.77-0.98)	.02	0.82 (0.72-0.93)	.001	0.99 (0.86-1.14)	.89
Counties in metropolitan areas of 250 000-999 999 population	0.91 (0.79-1.04)	.16	0.80 (0.69-0.91)	.001	1.34 (1.14-1.57)	<.001
Counties in metropolitan areas of 50 000-249 999 population	0.68 (0.53-0.89)	.005	0.60 (0.46-0.78)	<.001	1.06 (0.78-1.45)	.69
Micropolitan counties	0.96 (0.70-1.31)	.78	0.89 (0.65-1.22)	.46	1.38 (0.93-2.05)	.11
Not metropolitan or micropolitan counties	0.58 (0.37-0.89)	.01	0.53 (0.34-0.83)	.005	0.90 (0.53-1.51)	.68
Year (reference, 2016)						
2017	1.05 (0.88-1.25)	.61	1.06 (0.89-1.26)	.53	0.99 (0.81-1.20)	.90
2018	1.07 (0.89-1.27)	.48	1.10 (0.92-1.32)	.27	0.93 (0.76-1.13)	.44
2019	1.23 (1.04-1.47)	.02	1.31 (1.10-1.56)	.002	1.05 (0.86-1.27)	.63
2020	1.40 (1.18-1.68)	<.001	1.46 (1.22-1.74)	<.001	1.28 (1.04-1.57)	.02
2021	1.37 (1.15-1.63)	<.001	1.44 (1.21-1.72)	<.001	1.11 (0.91-1.35)	.30
2022	1.67 (1.41-1.98)	<.001	1.83 (1.54-2.17)	<.001	1.30 (1.08-1.58)	.007
Insurance (reference, private)						
Medicaid	1.58 (1.41-1.77)	<.001	1.39 (1.24-1.56)	<.001	1.79 (1.57-2.04)	<.001
Medicare	1.17 (0.96-1.44)	.01	1.07 (0.88-1.31)	.50	1.37 (1.03-1.82)	.03
No charge	1.97 (1.24-3.12)	.004	1.77 (1.10-2.84)	.02	2.90 (1.66-5.07)	<.001
Self-pay	1.97 (1.60-2.42)	<.001	1.77 (1.43-2.19)	<.001	2.17 (1.70-2.77)	<.001
Other	1.39 (1.07-1.80)	.01	1.30 (1.00-1.69)	.05	1.56 (1.16-2.09)	<.001
Hospital division (reference, Pacific)						
East North Central	1.03 (0.86-1.25)	.73	0.94 (0.77-1.13)	.50	1.31 (1.06-1.63)	.01
East South Central	1.11 (0.84-1.46)	.45	0.90 (0.68-1.19)	.47	1.78 (1.25-2.25)	.001
Middle Atlantic	0.84 (0.72-0.98)	.15	0.90 (0.76-1.05)	.18	0.77 (0.65-0.91)	.002
Mountain	1.62 (1.28-2.05)	<.001	1.52 (1.20-1.92)	<.001	2.03 (1.52-2.70)	<.001
New England	1.30 (1.03-1.63)	.03	1.47 (1.16-1.85)	.001	1.01 (0.78 to 1.32)	.92
South Atlantic	0.81 (0.69-0.95)	.01	0.78 (0.66-0.93)	.004	0.82 (0.68-0.98)	.03
West North Central	2.06 (1.51-2.81)	<.001	2.00 (1.45-2.75)	<.001	1.96 (1.35-2.86)	<.001
West South Central	0.82 (0.65-1.04)	.10	0.74 (0.58-0.94)	.01	1.02 (0.78-1.32)	.90
Teaching status (reference, rural)						
Urban nonteaching	5.28 (2.51-11.1)	<.001	6.06 (2.87-12.80)	<.001	3.94 (1.68-9.26)	.002
Urban teaching	7.13 (3.43-14.80)	<.001	8.63 (4.13-18.80)	<.001	4.57 (1.97-10.60)	<.001
Hospital bed size (reference, small)						
Medium	1.02 (0.87-1.20)	.08	1.04 (0.88-1.22)	.67	1.04 (0.87-1.25)	.67
Large	1.12 (0.97-1.29)	.12	1.16 (1.00-1.34)	.11	1.14 (0.97-1.34)	.11

Abbreviations: aOR, adjusted odd ratio; UFE, uterine fibroid embolization.

^a^ Other includes multiracial, unspecified, and groups not categorized under the predefined classification.

Compared with private insurance, patients with Medicaid (aOR, 1.58; 95% CI, 1.41-1.77), self-pay (aOR, 1.97; 95% CI, 1.60-2.42), and no-charge (aOR, 1.97; 95% CI, 1.24-3.12) were more likely to undergo UFE than surgical interventions, whereas patients enrolled in Medicare had higher utilization of UFE than myomectomy (aOR, 1.37; 95% CI, 1.03-1.82) (Table 2). Compared with patients living in the highest household income percentile, those in the lowest percentile were more likely to undergo UFE than myomectomy (aOR, 1.22; 95% CI, 1.04-1.43). Compared with patients living in dense metropolitan regions, those in rural regions were less likely to undergo UFE than hysterectomy (aOR, 0.53; 95% CI, 0.34-0.83) (Table 2).

### Age-Stratified Sensitivity Analysis

Among patients aged 30 to 39 years, Hispanic patients had lower odds of UFE than surgical interventions, and African American patients had lower odds of UFE than myomectomy. Among patients aged 40 to 49 years, African American patients had higher odds of undergoing UFE vs hysterectomy but lower odds compared with myomectomy. By age 50 years and older, UFE among African American patients was more likely than surgical interventions (eTable 2 in Supplement 1).

Patients with self-pay and Medicaid had higher odds of UFE across all age groups older than 30 years. Patients enrolled in Medicare had increased odds of undergoing UFE vs surgical interventions among patients aged 30 to 49 years, but lower odds of undergoing myomectomy among those aged 50 years or older (aOR, 0.55; 95% CI, 0.38-0.81). No-charge patients had higher odds of undergoing UFE than myomectomy among patients aged 30 to 49 years, and higher odds vs hysterectomy among those aged 40 to 49 years (eTable 3 in Supplement 1).

### Hospital Attributes and National Utilization Patterns

Compared with rural hospitals, urban nonteaching (aOR, 5.28; 95% CI, 2.51-11.10) and teaching (aOR, 7.13; 95% CI, 3.43-14.80) hospitals were more likely to perform UFE over surgical interventions. Hysterectomy was predominantly concentrated in rural, micropolitan counties with populations between 10 000 to 50 000, small hospitals, and urban nonteaching facilities, whereas UFE was concentrated in urban teaching, large hospitals, and central counties (Tables 1 and 2).

**Figure 2.**
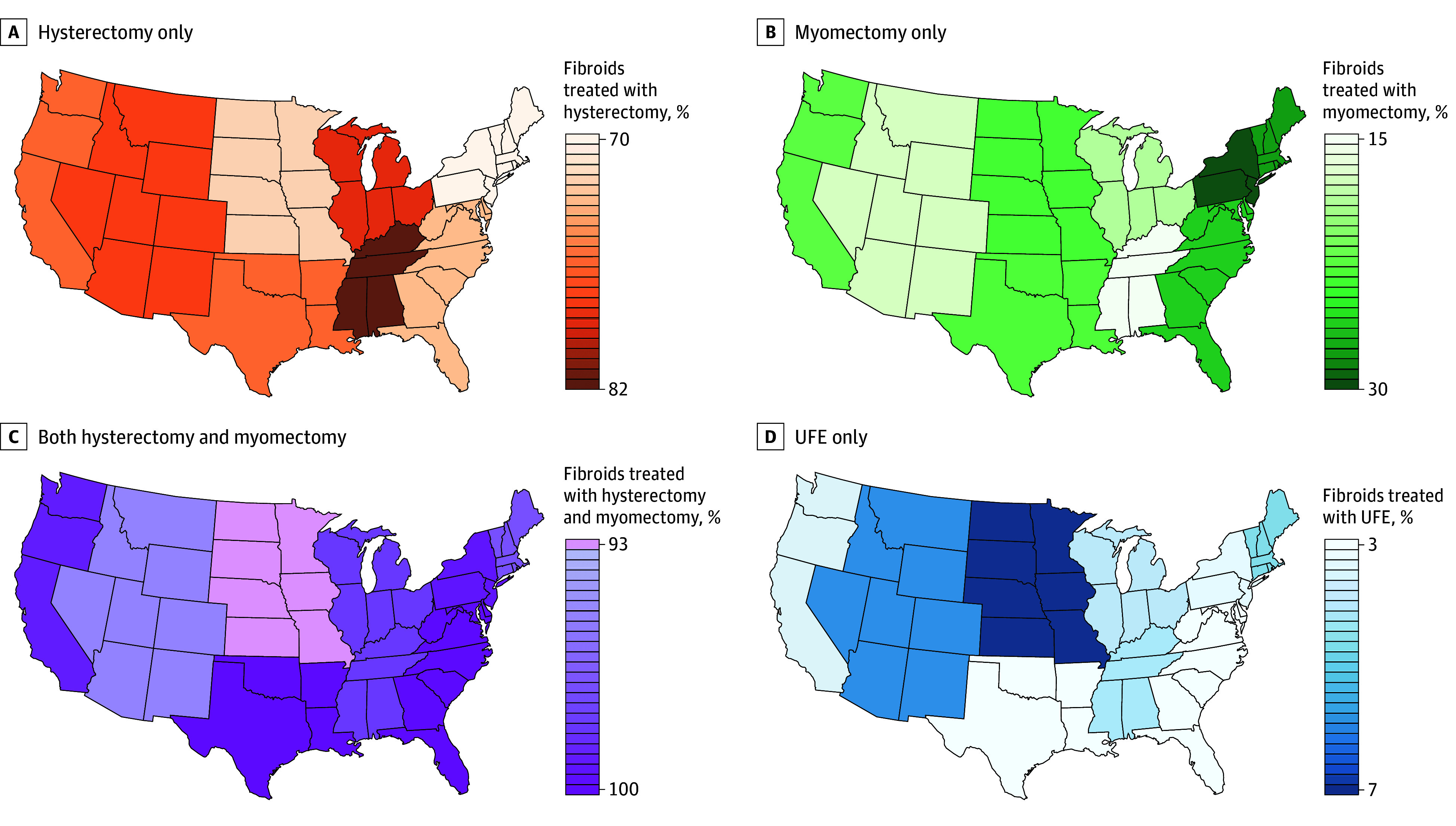
Regional Variation in Uterine Fibroid Treatment Modalities Across the US Graphs show utilization trends of hysterectomy only (A), myomectomy only (B), both hysterectomy and myomectomy (C), and uterine fibroid embolization (UFE) only (D). Hysterectomy rates were highest in the East South Central region, whereas myomectomy was most common in the Middle Atlantic and New England regions. Surgical interventions dominated overall in the South Atlantic region, and UFE use remained low nationwide, peaking in the West North Central and Mountain regions. Coastal regions (Pacific, New England, Middle Atlantic, and South Atlantic) tended to favor myomectomy more than central regions.

**Figure 3.**
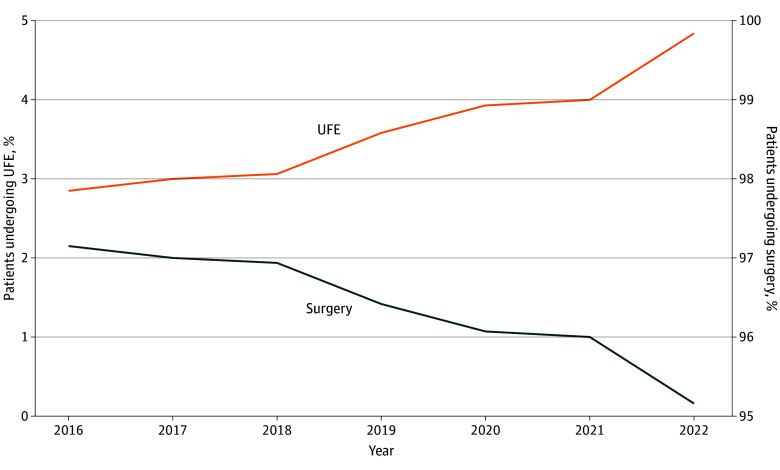
Time Trends of Inpatient Uterine Fibroid Embolization (UFE) and Surgical Interventions (Myomectomy and Hysterectomy) The percentage of UFE procedures increased over time, while the percentage of surgical procedures decreased.

Geographically, surgical procedures exceeded 95% in most regions, with the South Atlantic region having the highest rate (97.0%) and West North Central region having the lowest (93.1%) (Figure 2). The East South Central region had the highest rate for hysterectomy (81.5%), whereas New England (69.3%) and Middle Atlantic (69.0%) had the lowest. For myomectomy, the Middle Atlantic (27.7%) and New England (26.1%) regions had the highest rates, whereas the East South Central region had the lowest (14.4%). UFE utilization remained modest across all regions, with the highest rate in the West North Central region (6.9%) and the lowest in the South Atlantic region (3.0%). Compared with the Pacific region, the Mountain, New England, and West North Central regions had higher odds of UFE, whereas the Middle Atlantic and South Atlantic regions had lower odds of UFE (Table 2). Compared with 2016, patients in 2022 were more likely to undergo UFE than surgical interventions (aOR, 1.67; 95% CI, 1.41-1.98) (Table 2 and Figure 3).

## Discussion

This cross-sectional study presents a retrospective analysis of a nationally representative database to examine the utilization of procedural treatments for uterine fibroids and their association with patient demographics and hospital characteristics. Although some findings aligned with existing literature, the analysis shed light on how insurance and socioeconomic factors are associated with the likelihood of undergoing UFE vs surgical interventions. Notably, patients with Medicaid, Medicare, no-charge, or self-pay were more likely than those with private insurance to undergo UFE than surgical interventions. One potential explanation for this trend was discussed by Keller et al.^16^ Although the percentage of hysterectomies and myomectomies reimbursed by Medicaid or Medicare has remained stable over time, the reimbursement for UFE has recently increased, leading to a growing recognition of UFE among those payer groups. In addition, the lower cost of UFE may explain its higher utilization among self-pay and no-charge patients.^7^ However, patients with Medicaid were the second-largest insurance group in the fibroid cohort and accounted for a disproportionately higher share of UFE than surgical interventions. Those patients are often of a lower socioeconomic status, a trend that mirrors the income distribution, as those in the lowest household income percentile were more likely to undergo UFE than myomectomy. Conversely, privately insured patients, who represented most uterine fibroid cases, had a higher utilization of surgical interventions than UFE. This may reflect disparities in access associated with financial and institutional incentives, as well as referral networks, since gynecologists have to refer patients to IRs for UFE consultation or keep them for myomectomy or hysterectomy. In contrast, Medicare demonstrated a distinct pattern in UFE utilization. Among patients aged 30 to 49 years, who typically qualify for Medicare because of disability, UFE was performed more frequently than surgical interventions. This is likely because younger Medicare recipients often have disabling comorbid conditions that make a less-invasive option with UFE safer than surgery. However, paradoxically, among patients aged 50 years or older, UFE was less utilized than myomectomy, even though this older group typically has higher comorbidities and myomectomy is mostly performed in younger patients (median age, 37 years).

Although it is often assumed that UFE is primarily used by younger patients aiming to preserve fertility, this study found that the median age of patients undergoing UFE (45 years) was slightly lower than that of patients undergoing hysterectomy (47 years). Stratified analysis shows that among all fibroid-related encounters, nearly one-half were among patients aged 40 to 49 years, a group that also accounted for the majority of UFE and hysterectomy. Although few UFEs occurred in patients younger than 30 years, myomectomy predominated in this age group, and its utilization peaked among patients aged 30 to 39 years. In contrast, hysterectomy utilization increased with age, becoming the most common intervention among patients older than 40 years. Notably, increasing age was associated with lower odds of undergoing UFE vs hysterectomy, but higher odds of undergoing UFE vs myomectomy. This finding suggests that UFE serves as a transitional intermediate option between myomectomy in younger patients and hysterectomy in older patients. However, the higher utilization of hysterectomy and/or myomectomy, relative to the significant underuse of UFE, indicates missed opportunities to preserve the uterus and avoid surgical complications. Although age-based decision-making is appropriate in many cases, the study indicates that treatment selection is not solely associated with age, surgical risk, and fertility considerations, but also with insurance, income, practitioner preferences, and referral patterns. These raise concerns about disparities in how fibroid treatment options are offered, depending on a patient’s financial and social context. Such disparities suggest the possibility of preferential treatment patterns and referral biases associated with socioeconomic status, highlighting an area of concern that physicians and policymakers should address to enhance equity.

This study corroborates findings by Borah et al^4^ and Srinivas et al,^10^ who similarly reported increased odds of African American patients undergoing UFE vs hysterectomy. This could be attributed to the evidence that African American patients face a 3-fold increased risk of symptomatic fibroids and are more likely to experience complications.^17^ However, this study found that White patients have the highest likelihood of hysterectomy. In the US, White patients are generally economically more advantaged with higher rates of private insurance.^18^ This may suggest a utilization pattern in which certain physicians are more likely to recommend surgery for economically advantaged groups and UFE for others. Furthermore, potential referral bias and limited access to IRs may contribute to the predominance of hysterectomy, especially in rural and suburban regions. However, by restricting the analysis to hospitals that offered all 3 procedures, the study minimized the impact of procedural availability, suggesting that differences in referral patterns, institutional norms, or patient preferences likely explain the disparity. Conversely, UFE was more common in patients living in central counties and seeking care at urban hospitals.^19^ This may reflect the role of academic medical centers, which typically offer advanced procedures like UFE.^20,21^ Since these centers are often located in low-income urban areas and serve a large publicly insured population, this could confound the observed association between Medicaid, low income, and higher UFE utilization. However, the analysis also reveals that hysterectomy remains the predominant treatment in rural and urban nonteaching hospitals. This emphasizes the need for multidisciplinary collaborative decision-making between gynecologists and IRs to make the appropriate treatment recommendations and influence the patient population being referred for the most appropriate procedure for superior patient outcomes. In addition, there is a need for tools to enhance patient education and literacy about uterine fibroid treatment options for more informed decisions.

Other studies also demonstrated a decline in surgical treatments for fibroids,^22^ with a growing trend toward more UFE, likely owing to the financial burden of surgery.^7,22,23^ Although UFE offers effective symptom management and shorter hospital stays,^1,24^ it remains significantly underutilized. One reason is that there are limited and conflicting data about fertility outcomes after UFE compared with myomectomy.^25^ Second, limited awareness about the benefits and risks of UFE among gynecologists leads to recommendations for surgical interventions.^25^ This is also reflected in public interest in UFE, which lags far behind myomectomy as measured by volumes of public online search and publications.^26^ Collaboration between gynecologists and IRs may increase UFE utilization, as supported by a study featuring a multidisciplinary fibroid clinic in an urban health care system that included IRs and gynecologists. The clinic led to an increase in UFE referrals.^27^ However, referrals may still be inherently limited for patients with large or multiple fibroids that are not suitable for UFE.^20^ This could result from delayed presentation, which is more common among publicly insured or underrepresented patients, for whom myomectomy or hysterectomy becomes the only viable option. Thus, standardizing IR protocols for UFE and fostering multidisciplinary collaboration and communication with gynecologists are essential strategies to improve early referrals and broaden the pool of patients considered for UFE.

### Limitations

This study has limitations that should be mentioned. Recently, there has been a shift from admission following UFE to performing outpatient UFE. Medications have been optimized to better control postprocedural symptoms.^28^ Although this study uses a national database, 1 limitation is that it only includes inpatient data and lacks outpatient UFE. Generally, the inpatient population is more complex, necessitating a hospital setting for their procedures; as such, this study allows understanding of the full spectrum of UFE. Wang et al^7^ investigated utilization of UFE, myomectomy, and hysterectomy in the outpatient setting across 2 US states. Unlike the present study, they found an increasing proportion of outpatient hysterectomy than UFE.^7^ These incongruent results highlight that differences in cost and availability of fibroid procedures in the inpatient vs outpatient setting may play a role in utilization trends, which serves as an interesting future research direction. Nationally, HCUP is the largest longitudinal health care database in the US.^29^ However, outpatient UFEs are not captured by HCUP Nationwide Ambulatory Surgery Sample, which makes it challenging to do a longitudinal nationwide analysis of outpatient UFEs. Such analysis can be conducted only through the State Ambulatory Surgery and Services Database, which has individual yearly state databases. However, owing to the high level of hospital identifiability in those state databases, HCUP imposes restrictions and costs to access them, thus limiting the ability to achieve a nationally comprehensive representation of outpatient fibroid procedures. This could be streamlined through our societies to advocate for HCUP to provide access to these datasets, allowing analysis of nationwide trends of outpatient UFE. This would enable analysis of the geographical distribution of UFE compared with other fibroid services, allowing evaluation of how practitioners’ location influences UFE utilization. It is also important to advocate for the inclusion of outpatient UFE in Nationwide Ambulatory Surgery Sample, enabling nationwide analyses without relying on fragmented, state-specific databases. This would support ongoing monitoring of trends to ensure that policy efforts addressing disparities in fibroid management are implemented effectively and equitably across both outpatient and inpatient settings.

Another limitation is the reliance on billing codes, which may be subject to inaccuracies. The study overlaps with the COVID-19 pandemic, during which hospital capacity limitations led to delayed procedures. Although UFE utilization increased from 2016 to 2020, there was a decline in 2021, coinciding with an increase in myomectomies, suggesting a possible shift to surgery. A rebound was observed in 2022, which may reflect postponed procedures. Besides that, future studies would benefit from incorporating clinical appropriateness criteria using variables like fibroid size, symptoms, and fertility desire, variables that are not available in the HCUP. Additionally, 4% of records were excluded due to missing demographic or hospital-level variables, which may introduce sampling bias. Future studies can consider imputation methods to address this. In addition, despite multivariable analysis, residual confounding may still influence the results.

## Conclusions

In this cross-sectional study, patients older than 40 years, African American patients, those enrolled in Medicaid or Medicare or who were no-charge or self-pay, and those living in lower-income communities were more likely to undergo UFE vs surgical uterine fibroid treatments. Patients at rural hospitals, White patients, and those with private insurance were more likely to undergo surgical interventions. Although utilization of UFE procedures has increased, they still lag far behind surgery. The study encourages efforts to increase public awareness of UFE and improve its access equitably across the nation, ensuring that all patients receive fair, consistent, and high-quality care for uterine fibroids.

## Notes

Supplement 1. **eTable 1.** *10th Revision of the International Statistical Classification of Diseases and Related Health Problems (ICD-10)* Codes Used in the Study **eTable 2.** Stratified Odd Ratios By Age for Racial/Ethnic Groups **eTable 3.** Stratified Odd Ratios by Age for Insurance Status **eFigure 1.** A Nationwide Analysis of Uterine Fibroid Management Using the HCUP Database to Compare UFE to Surgical Interventions (Myomectomy and Hysterectomy) Across the US **eFigure 2.** Heat Map Showing the Proportion of Insurance Status Across Various Racial Groups (i.e Percentages From n-by-n Table)

Supplement 2.
Data Sharing Statement
