# Supplementary material for: Disparities in Utilization of Uterine Fibroid Embolization
Source: JAMA Netw Open. 2025 Sep 16;8(9):e2532100. doi: 10.1001/jamanetworkopen.2025.32100 (PMC12441870; doi:10.1001/jamanetworkopen.2025.32100)
Supplement: Supplement 1. — eTable 1. 10th Revision of the International Statistical Classification of Diseases and Related Health Problems (ICD-10) Codes Used in the Study eTable 2. Stratified Odd Ratios By Age for Racial/Ethnic Groups eTable 3. Stratified Odd Ratios by Age for Insurance Status eFigure 1. A Nationwide Analysis of Uterine Fibroid Management Using the HCUP Database to Compare UFE to Surgical Interventions (Myomectomy and Hysterectomy) Across the US eFigure 2. Heat Map Showing the Proportion of Insurance Status Across Various Racial Groups (i.e Percentages From n-by-n Table) [file jamanetwopen-e2532100-s001.pdf]

Supplemental Online Content

Elhakim TS, Smolinski-Zhao S, Miyasato D, et al. Disparities in utilization of uterine fibroid embolization. *JAMA Netw Open*. 2025;8(9):e2532100.
doi:10.1001/jamanetworkopen.2025.32100

eTable 1. *10th Revision of the International Statistical Classification of Diseases and Related Health Problems (ICD-10)* Codes Used in the Study
eTable 2. Stratified Odd Ratios By Age for Racial/Ethnic Groups
eTable 3. Stratified Odd Ratios by Age for Insurance Status
eFigure 1. A Nationwide Analysis of Uterine Fibroid Management Using the HCUP Database to Compare UFE to Surgical Interventions (Myomectomy and Hysterectomy) Across the US
eFigure 2. Heat Map Showing the Proportion of Insurance Status Across Various Racial Groups (i.e Percentages From n-by-n Table)

This supplemental material has been provided by the authors to give readers additional information about their work.

© 2025 Elhakim TS et al. *JAMA Network Open*.

eTable 1. ***10th Revision of the International Statistical Classification of Diseases and Related Health Problems (ICD-10)* Codes Used in the study**

**Diagnosis**	***ICD-10-CM* codes**
Submucous leiomyoma of uterus	D25.0
Intramural leiomyoma of uterus	D25.1
Subserosal leiomyoma of uterus	D25.2
Leiomyoma of uterus, unspecified	D25.9

**Procedure**	***ICD-10-PCS* codes**
Uterine Fibroid Embolization	04LE3ZT, 04LF3DU, 04LF3ZU, 04LE3DT
Hysterectomy	0UT90ZZ, 0UT94ZZ, 0UT97ZZ, 0UT98ZZ, 0UT9FZZ, 0UT97ZL, 0UT98ZL, 0UT90ZL, 0UT94ZL, 0UT9FZL
Myomectomy	0UB90ZZ, 0UB93ZZ, 0UB94ZZ, 0UB97ZZ, 0UB98ZZ

CM= Diagnosis PCS=Procedure

© 2025 Elhakim TS et al. *JAMA Network Open*.

eTable 2. Stratified Odd Ratios By Age for Racial/Ethnic Groups

Racial/Ethnic Group (Ref: Whites)	Procedural Comparison	Age <30	Age 30 - 39	Age 40 - 49	Age 50+
Asian or Pacific Islander	UFE vs Surgical Interventions	1.94 (0.57-6.62), p=0.29	1.01 (0.65-1.57), p=0.97	1.07 (0.80-1.44), p=0.64	0.80 (0.46-1.36), p=0.41
	UFE vs Hysterectomy	9.26 (0.81-106), p=0.07	1.46 (0.90-2.36), p=0.12	1.12 (0.83-1.51), p=0.44	0.80 (0.47-1.37), p=0.42
	UFE vs Myomectomy	1.81 (0.54-6.02), p=0.33	0.79 (0.50-1.24), p=0.30	0.78 (0.56-1.07), p=0.13	0.74 (0.37-1.47), p=0.39
African American	UFE vs Surgical Interventions	0.62 (0.26-1.50), p=0.29	0.81 (0.62-1.06), =0.12	1.48 (1.24-1.76), p=<0.001	1.92 (1.48-2.48), p=<0.001
	UFE vs Hysterectomy	1.20 (0.34-4.17), p=0.78	1.20 (0.91-1.60), p=0.20	1.68 (1.41-2.00), p<0.001	1.92 (1.48-2.48), p<0.001
	UFE vs Myomectomy	0.56 (0.23-1.35), p=0.20	0.62 (0.47-0.81), p<0.001	0.74 (0.61-0.90), p=0.003	2.04 (1.44-2.88), p<0.001
Hispanic	UFE vs Surgical Interventions	0.76 (0.25-2.29), p=0.63	0.65 (0.47-0.90), p=0.01	0.83 (0.67-1.03), p=0.09	0.92 (0.64-1.32), p=0.64
	UFE vs Hysterectomy	0.51 (0.11-2.40), p=0.39	0.66 (0.47-0.92), p=0.01	0.84 (0.67-1.04), p=0.11	0.91 (0.64-1.31), p=0.63
	UFE vs Myomectomy	0.75 (0.25-2.25), p=0.60	0.65 (0.47-0.90), p=0.01	0.75 (0.59-0.96), p=0.02	0.99 (0.61-1.60), p=0.96

© 2025 Elhakim TS et al. *JAMA Network Open*.

**Native American**	UFE vs Surgical Interventions	n/a	0.55 (0.07-4.18), p=0.56	1.89 (0.86-4.14), p=0.11	n/a
	UFE vs Hysterectomy	n/a	0.60 (0.08-4.64), p=0.63	1.90 (0.86-4.18), p=0.11	n/a
	UFE vs Myomectomy	n/a	0.65 (0.08-5.13), p=0.68	1.69 (0.60-4.78), p=0.33	n/a
**Other**	UFE vs Surgical Interventions	1.64 (0.48-5.59), p=0.43	0.76 (0.47-1.23), p=0.27	1.07 (0.78-1.46), p=0.67	1.12 (0.65-1.91), p=0.69
	UFE vs Hysterectomy	2.72 (0.33-22.7), p=0.35	1.0 (0.60-1.64), p=0.98	1.18 (0.86-1.62), p=0.30	1.12 (0.65-1.92), p=0.68
	UFE vs Myomectomy	1.53 (0.45-5.27), p=0.50	0.66 (0.40-1.07), p=0.09	0.61 (0.43-0.85), p=0.004	1.10 (0.54-2.24), p=0.79

© 2025 Elhakim TS et al. *JAMA Network Open*.

eTable 3. Stratified Odd Ratios by Age for Insurance Status

Insurance Status (Ref: Private insurance)	Procedural Comparison	Age <30	Age 30 - 39	Age 40 - 49	Age 50+
**Medicaid**	UFE vs Surgical Interventions	0.76 (0.32-1.78), p=0.52	1.83 (1.46-2.29), p=<0.001	1.52 (1.30-1.77), p=<0.001	1.66 (1.28-2.17), p<0.001
	UFE vs Hysterectomy	0.34 (0.11-1.04), p=0.06	1.25 (0.99-1.57), p=0.066	1.46 (1.25-1.71), p<0.001	1.70 (1.30-2.22), p<0.001
	UFE vs Myomectomy	0.83 (0.35-1.95), p=0.66	2.44 (1.93-3.08), p<0.001	1.78 (1.48-2.13), p<0.001	0.97 (0.67-1.41), p=0.87
**Medicare**	UFE vs Surgical Interventions	n/a	2.37 (1.38-4.08), p=0.002	2.00 (1.45-2.75), p=<0.001	0.81 (0.62-1.08), p=0.15
	UFE vs Hysterectomy	n/a	1.37 (0.79-2.37), p=0.26	1.87 (1.35-2.58), p<0.001	0.83 (0.63-1.09), p=0.18
	UFE vs Myomectomy	n/a	3.76 (2.09-6.75), p<0.001	2.93 (1.96-4.39), p<0.001	0.55 (0.38-0.81), p=0.002
**No charge**	UFE vs Surgical Interventions	n/a	2.46 (0.88-6.90), p=0.09	2.10 (1.17-3.75), p=0.01	1.55 (0.48-5.02), p=0.47
	UFE vs Hysterectomy	n/a	1.77 (0.60-5.22), p=0.30	1.95 (1.09-3.52), p=0.03	1.53 (0.47-4.96), p=0.48
	UFE vs Myomectomy	n/a	3.11 (1.08-8.97), p=0.04	3.34 (1.62-6.89), p=0.001	2.47 (0.40-15.3), p=0.33

© 2025 Elhakim TS et al. *JAMA Network Open*.

Other	UFE vs Surgical Interventions	2.89 (0.83-10.1), p=0.10	0.86 (0.44-1.68), p=0.66	1.63 (1.18-2.27), p=0.003	1.06 (0.55-2.04), p=0.86
	UFE vs Hysterectomy	4.47 (0.30-65.9), p=0.27	0.71 (0.36-1.40), p=0.32	1.57 (1.13-2.19), p=0.007	1.06 (0.55-2.03), p=0.87
	UFE vs Myomectomy	3.30 (0.97-11.3), p=0.06	0.95 (0.48-1.87), p=0.89	1.78 (1.19-2.65), p=0.005	1.32 (0.48-3.64), p=0.59
Self-pay	UFE vs Surgical Interventions	1.60 (0.31-8.37), p=0.58	1.69 (1.06-2.72), p=0.03	2.00 (1.52-2.64), p<0.001	2.29 (1.49-3.52), p<0.001
	UFE vs Hysterectomy	1.21 (0.12-12.7), p=0.87	1.17 (0.71-1.91), p=0.54	1.94 (1.46-2.56), p<0.001	2.33 (1.51-3.58), p<0.001
	UFE vs Myomectomy	1.71 (0.30-9.67), p=0.54	2.20 (1.36-3.57), p=0.001	2.48 (1.78-3.47), p<0.001	1.37 (0.72-2.61), p=0.34

© 2025 Elhakim TS et al. *JAMA Network Open*.

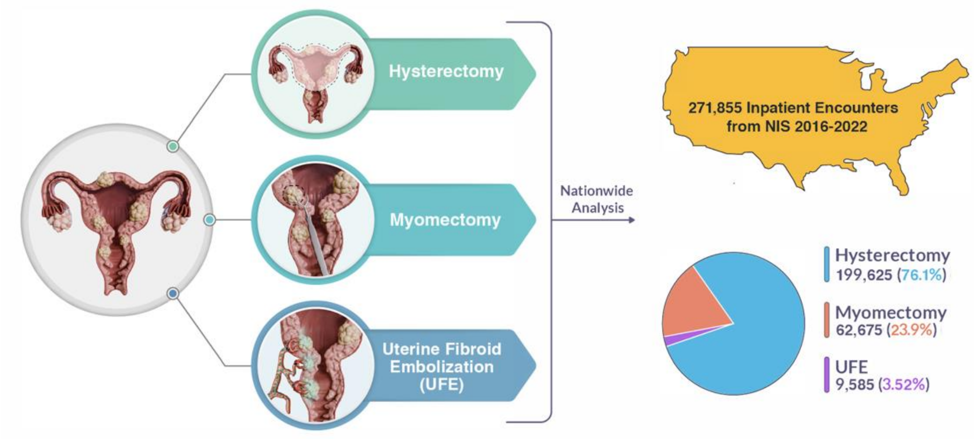

eFigure 1. A Nationwide Analysis of Uterine Fibroid Management Using the HCUP Database to Compare UFE to Surgical Interventions (Myomectomy and Hysterectomy) Across the US.

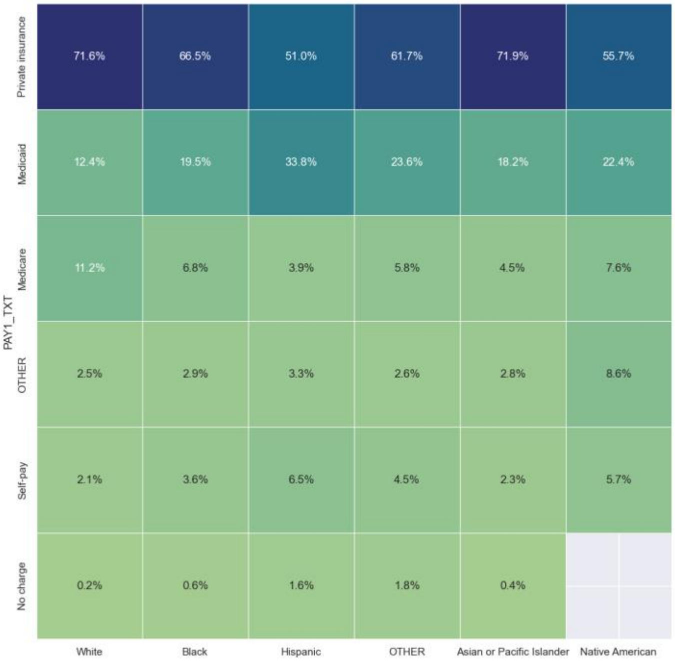

eFigure 2: Heat Map Showing the Proportion of Insurance Status Across Various Racial Groups (i.e Percentages From n-by-n Table)

© 2025 Elhakim TS et al. *JAMA Network Open*.
