# Supplementary material for: Disparities in Utilization of Uterine Fibroid Embolization
Source: JAMA Netw Open. 2025 Sep 16;8(9):e2532100. doi: 10.1001/jamanetworkopen.2025.32100 (PMC12441870; doi:10.1001/jamanetworkopen.2025.32100)
Supplement: Supplement 2. — Data Sharing Statement [file jamanetwopen-e2532100-s002.pdf]

Data Sharing Statement

Elhakim. Disparities in Utilization of Uterine Fibroid Embolization. *JAMA Netw Open*. Published September 16, 2025. doi:10.1001/jamanetworkopen.2025.32100

Data

Data available: No

Additional Information

Explanation for why data not available: The data used in this study were derived from the HCUP Nationwide Inpatient Sample (NIS), a publicly available all-payer database. Data is contingent on HCUP data use agreement compliance.
